# Timeless noncoding DNA contains cell-type preferential enhancers important for proper *Drosophila* circadian regulation

**DOI:** 10.1073/pnas.2321338121

**Published:** 2024-04-03

**Authors:** Dingbang Ma, Pranav Ojha, Albert D. Yu, Maisa S. Araujo, Weifei Luo, Evelyn Keefer, Madelen M. Díaz, Meilin Wu, William J. Joiner, Katharine C. Abruzzi, Michael Rosbash

**Affiliations:** ^a^Interdisciplinary Research Center on Biology and Chemistry, Shanghai Institute of Organic Chemistry, Chinese Academy of Sciences, Shanghai 201210, China; ^b^Shanghai Key Laboratory of Aging Studies, Shanghai 201210, China; ^c^HHMI, Brandeis University, Waltham, MA 02453; ^d^Department of Biology, Brandeis University, Waltham, MA 02453; ^e^Laboratory of Entomology, Fiocruz Rondônia and Programa de Pós-Graduação em Biologia Experimental/Programa Nacional de Pós-Doutorado, Federal University Foundation of Rondônia, Porto Velho 76801-974, Brazil; ^f^Guangxi Academy of Sciences, Nanning 530003, China; ^g^The Miami Project to Cure Paralysis, Department of Neurosurgery, University of Miami Miller School of Medicine, Miami, FL 33136; ^h^Department of Pharmacology, University of California, San Diego, La Jolla, CA 92093; ^i^Center for Circadian Biology, University of California, San Diego, La Jolla, CA 92093

**Keywords:** circadian, clock neurons, gene regulation

## Abstract

To address the contribution of transcriptional regulation to *Drosophila* clock gene expression and to behavior, we generated a series of CRISPR-mediated deletions within two regions of the circadian gene *timeless* (*tim*), an intronic E-box region and an upstream E-box region that are both recognized by the key transcription factor Clock (Clk) and its heterodimeric partner Cycle. The upstream deletions but not an intronic deletion dramatically impact *tim* expression in fly heads; the biggest upstream deletion reduces peak RNA levels and *tim* RNA cycling amplitude to about 15% of normal, and there are similar effects on *tim* protein (TIM). The cycling amplitude of other clock genes is also strongly reduced, in these cases due to increases in trough levels. These data underscore the important contribution of the upstream E-box enhancer region to *tim* expression and of TIM to clock gene transcriptional repression in fly heads. Surprisingly, *tim* expression in clock neurons is only modestly affected by the biggest upstream deletion and is similarly affected by a deletion of the intronic E-box region. This distinction between clock neurons and glia is paralleled by a dramatically enhanced accessibility of the intronic enhancer region within clock neurons. This distinctive feature of *tim* chromatin was revealed by ATAC-seq (assay for transposase-accessible chromatin with sequencing) assays of purified neurons and glia as well as of fly heads. The enhanced cell type–specific accessibility of the intronic enhancer region explains the resilience of clock neuron *tim* expression and circadian behavior to deletion of the otherwise more prominent upstream *tim* E-box region.

## Significance

Drosophila circadian rhythms are governed by a feedback loop, in which the positive transcription factor CLK (CLOCK)/CYC (CYCLE) associates with two E-box–containing enhancers, one upstream and one intronic, within its negative regulatory gene timeless. Reporter gene experiments from more than 20 y ago showed that only the upstream enhancer affects timeless expression in tissue culture and fly heads. Consistent with these observations are current experiments indicating that the upstream enhancer is essential for timeless expression in glia. However, these deletions only modestly affect clock neuron timeless expression and circadian behavior. ATAC-seq experiments show that the intronic enhancer is much more active in neurons than glia, which indicate a discrete regulatory program for core clock genes in circadian neurons.

The circadian clock is an evolutionarily conserved, adaptive feature of many eukaryotic organisms. It confers fitness by a daily adjustment of its core molecular pacemaker to the precise 24 h environmental fluctuations of light/dark cycles, temperature, or nutrition (1). The pacemaker drives a broad sweep of rhythmic gene expression, which plays a central role in animal physiology, metabolism, and behavior (2–6). The diversity of gene expression rhythms reflects the fact that the core circadian machinery ticks away in many if not most cells and tissues, including in a wide expression pattern across the mammalian brain (7–9). Nonetheless, the molecular pacemaker is most notable—perhaps strongest—in a restricted region, the suprachiasmatic nucleus (SCN); these approximately 20,000 hypothalamic neurons sit directly above the optic chiasm and play an important role in circadian physiology (10). There is a comparably important circadian region in the fly brain, the ca. 150 *Drosophila* clock neurons (11, 12), and there is evidence that both these regions have special properties: For example, their anatomy and/or gene expression profiles allow the perception of the external light–dark cycle, in contrast to most of the brain (13, 14).

This core circadian pacemaker is based on a now classical transcription-translation feedback loop, which generates ~24 h periodicity (15, 16). A somewhat simplified description of this pacemaker in mammals is as follows. The transcription factor circadian locomotor output cycles kaput (CLOCK) and Basic helix-loop-helix ARNT-like protein 1 (BMAL1) activates the transcription of two key direct target genes, *Period* (*Per*) and *Cryptochrome* (*Cry*). PER and CRY then accumulate and eventually function as transcriptional repressors, including the recruitment of corepressors to negatively regulate CLOCK/BMAL1 activity (15). The noncanonical E-box and a B-site (a bzip transcription factor E4BP4 binding site) are indispensable for the activation and repression of *Period 2* (17, 18). In addition, daytime elements (D box) and nighttime elements of the *Cry* regulatory region fine tunes its expression to maintain rhythmicity (19). When the negative regulators decay after about 24 h, a new cycle begins.

A similar picture exists in *Drosophila*. The transcription factors Clock (CLK) and cycle (CYC) form a heterodimer and are the fly orthologs of CLOCK/BMAL1 (20, 21). CLK/CYC directly activates *per* and *timeless* (*tim*) expression by binding to their E-box enhancer elements (22–24). PER and TIM, the products of *per* and *tim*, accumulate during the night, enter the nucleus, and eventually suppress the transcriptional activity of CLK/CYC, like the suppression of CLOCK/BMAL1 by PER and CRY in mammals. PER and TIM are then degraded in the morning, releasing CLK/CYC to initiate a new cycle (25, 26).

There are two canonical E-box regions in *tim* regulatory DNA; one is located upstream of the promoter and the other is within the large first intron. A previous study showed that the intronic E-box makes no contribution to *tim* expression from transgenic reporter genes, in S2 cells as well as in fly heads. In contrast, the upstream E-box elements along with two adjacent elements, a noncanonical E-box element and a somewhat enigmatic PER-box, are necessary for proper expression of these reporter gene constructs, also in fly heads and in S2 cells (27). This upstream E-box region is evolutionarily conserved in insect species, consistent with the notion that it is essential for the circadian transcriptional regulation of *tim* (28). There are other gene expression analyses, reporter gene studies, chromatin immunoprecipitation (ChIP) assays, and missense mutations in key circadian transcription factors, all of which are consistent with an important role of this region and its regulatory motifs to circadian rhythmicity (23, 27, 29). There are similar studies in mammals. For example, the E-box–mediated core feedback loop is interlocked with the RRE-mediated feedback loop, but deleting the RRE motif in the *Bmal1* gene results in apparent normal circadian rhythms (30). Notable in this context is that there are no simple *cis*-regulatory mutations showing that these elements are essential for circadian rhythmicity in the fly.

In this study, we used CRISPR/Cas9 to generate a series of deletions in the E-box elements of both the upstream and the intronic regions of *tim*. Not unexpectedly, *tim* mRNA and protein abundance in heads was dramatically reduced in the upstream mutant strains. The data indicate that this E-box containing promotor-proximal region contributes to *tim* transcription and also that TIM contributes to clock gene repression. Surprisingly however, even the largest deletion had no more than a modest effect on locomotor activity rhythms despite a very strong effect on clock gene expression in heads. This disconnect suggested a substantial difference between the impact of the deletion on clock neurons and on other circadian tissues. Indeed, in situ hybridization and immunocytochemistry assays show that the clock neurons are substantially resistant to the effects of even the biggest upstream deletion compared to other cells and tissues. Distinct chromatin accessibility profiles from sorted neurons vs. heads and glia offer an attractive explanation for this resistance. The data indicate a feature of transcriptional regulation specific to fly clock neurons and also provide direct genetic evidence to support the importance of a key *cis*-regulatory element to circadian rhythm regulation.

## Results

### Fly Head Clock Gene Expression in Deletion Mutants of *tim* Regulator DNA.

**Fig. 1.**
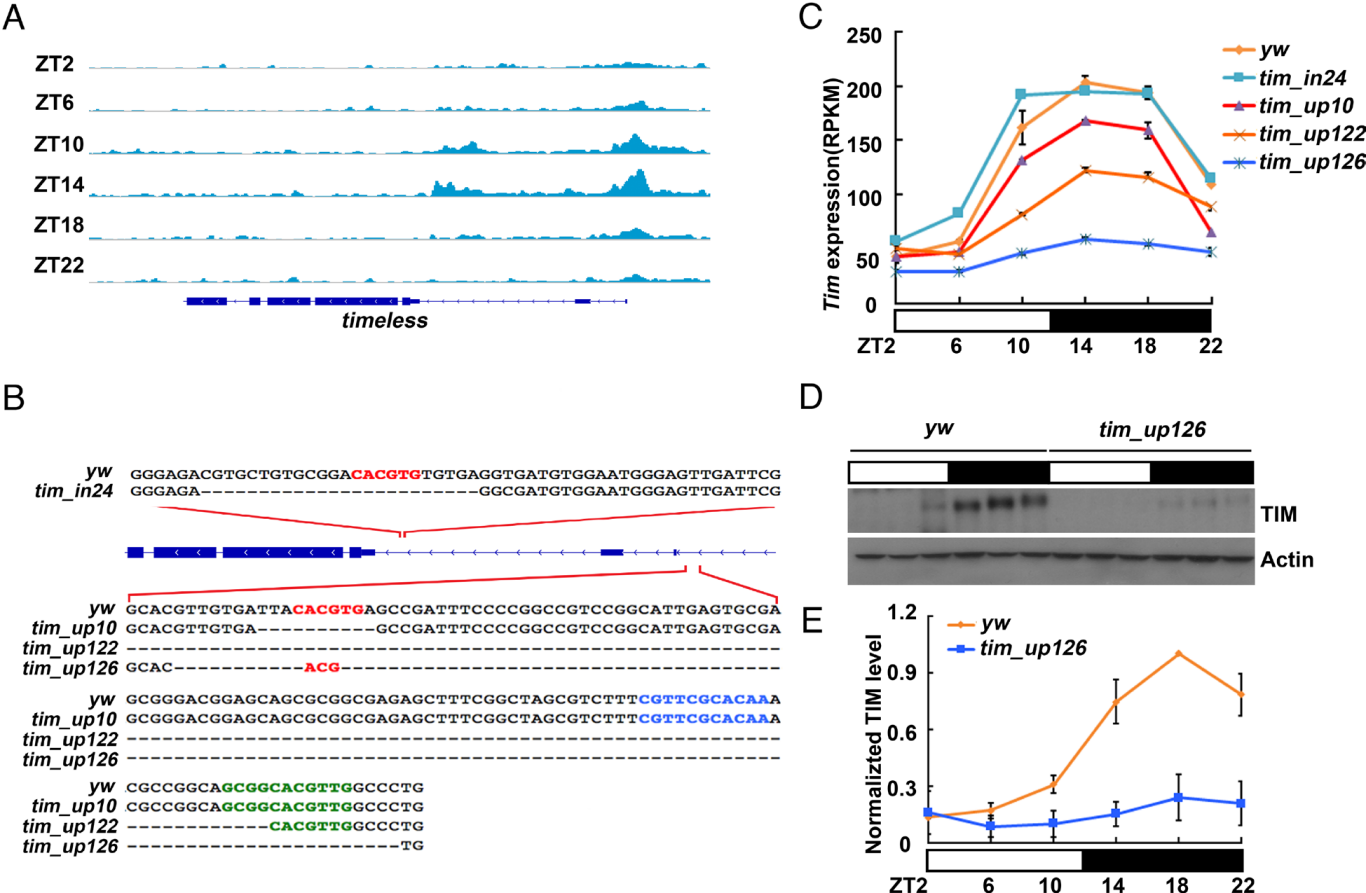
Generation of deletion mutants in the promoter region of *tim* by CRISPR-cas9. (*A*) ChIP signal of CLK binding at *tim* in wild-type flies. CLK binds in two distinct E-box containing regions of the *tim* promoter: upstream of the transcriptional start site and within the second intron. CLK binding changes throughout the day peaking at ZT14. The direction of transcription is *Right* to *Left*. The *y*-axis scale is identical for all time points. (*B*) Description of *tim* promoter mutants generated by CRISPR-cas9. The deletion mutants are aligned to the wild-type (*yw*) genomic sequence. E-boxes are labeled in red, PER-boxes are labeled in blue, and TER-boxes are labeled in green. The deletions were named according to their position and size: *tim_in24* (e.g., 24 bp deletion in the intron), *tim_up10 (*e.g., 10 bp deletion in the upstream region), *tim_up122* and *tim_up126.* (*C*) RNA-sequencing results from fly heads show that there is a steady decrease in *tim* expression as the promoter deletions progress from mild to more extensive. Flies were collected at six timepoints throughout the day. Error bars represent the SD of two biological replicates. For *tim_in24*, *tim_up10*, we did one replication because of they only have very marginal effect on *tim* expression. The *y*-axis shows RPKM values (Reads Per Kilobase of transcript per Million mapped reads) normalized to the maximal level of mRNA during the day. (*D*) A representative western blot showing that TIM is significantly reduced in the *tim_up126* compared to the wild-type control (*yw*). Flies were collected at six time points throughout the day. Beta-actin was used as a loading control. (*E*) Quantification of TIM levels in *yw* and *tim_up126* as shown in *D*. TIM levels were normalized to beta-actin. Error bars represent the SD of two biological repeats.

To decrease the expression of *tim* mRNA, CRISPR-mediated deletions were made in the two regulatory regions known to affect *tim* reporter gene expression: the region upstream of the transcription start site and a region within the first intron of the *tim* gene. Both regions include E-box, which almost certainly help recruit the CLK:CYC complex to the chromatin (Fig. 1*A*). The upstream regulatory region is larger and more complicated than the intronic region as the former contains not only E-boxes but also E-box–like motifs (27, 31).

In the intronic region, a 24 bp deletion resulted (*tim_in24*) in a deletion of intronic E-box. In the upstream region, we obtained three useful deletions of 10 bp, 122 bp, and 126 bp: *tim_up10*, *tim_up122*, and *tim_up126*, respectively (Fig. 1*B*). They all removed the E-box, and the two larger deletions also removed the other two E-box–like motifs within this region. The 2nd chromosomes containing these deletions were made homozygous for subsequent biochemical and behavioral analyses. It is worth noting that a canonical E-box was recreated by accident in the *tim_up126* deletion; it is ~14 bp upstream of the position of the endogenous E-box (Fig. 1*B*).

The intronic deletion *tim_in24* had little or no effect on *tim* gene expression (Fig. 1*C*, yellow vs. teal). In contrast, all deletions in the upstream region decreased *tim* expression compared to control flies (see Fig. 1*C* for the smallest 10 bp *tim_up10* deletion vs. WT, yellow vs. red). The two larger upstream deletions had even bigger effects (Fig. 1*C* blue, orange). The data are completely consistent with previous studies using reporter gene assays, which showed that deletions of the upstream E-box are much more important for transcription activation than the intronic E-box (27, 31). Notably, the cycling of *tim* mRNA expression from the deletion strains appeared largely normal except for its lower amplitude, an effect that is largely if not exclusively due to reduced peak levels with little or no change of trough levels. We also carried out qRT-PCR to assess the expression level of *tim* in these mutants with similar results (*SI Appendix*, Fig. S1). Not surprisingly, the biggest effect was from the largest E-box containing deletion, *tim_up126*, which expressed about 15% of WT *tim* mRNA levels (Fig. 1*C*). This strain also expressed comparably low levels of TIM protein (Fig. 1 *D* and *E*).

These deletion strains appear to constitute an allelic series: *tim* gene expression progressively decreases, presumably reflecting reduced binding of the positive transcription factor CLK:CYC to this important upstream region (*Discussion*). As the role of TIM in gene expression regulation is less well-characterized than that of PER, we wondered how the reduced TIM levels would affect the expression of other clock genes that are also direct targets of the CLK:CYC complex. To this end, *vrille* (*vri*), *per*, and *PAR domain protein 1 epsilon* (*pdp1*) expression was carefully assessed in the deletion strain series.

**Fig. 2.**
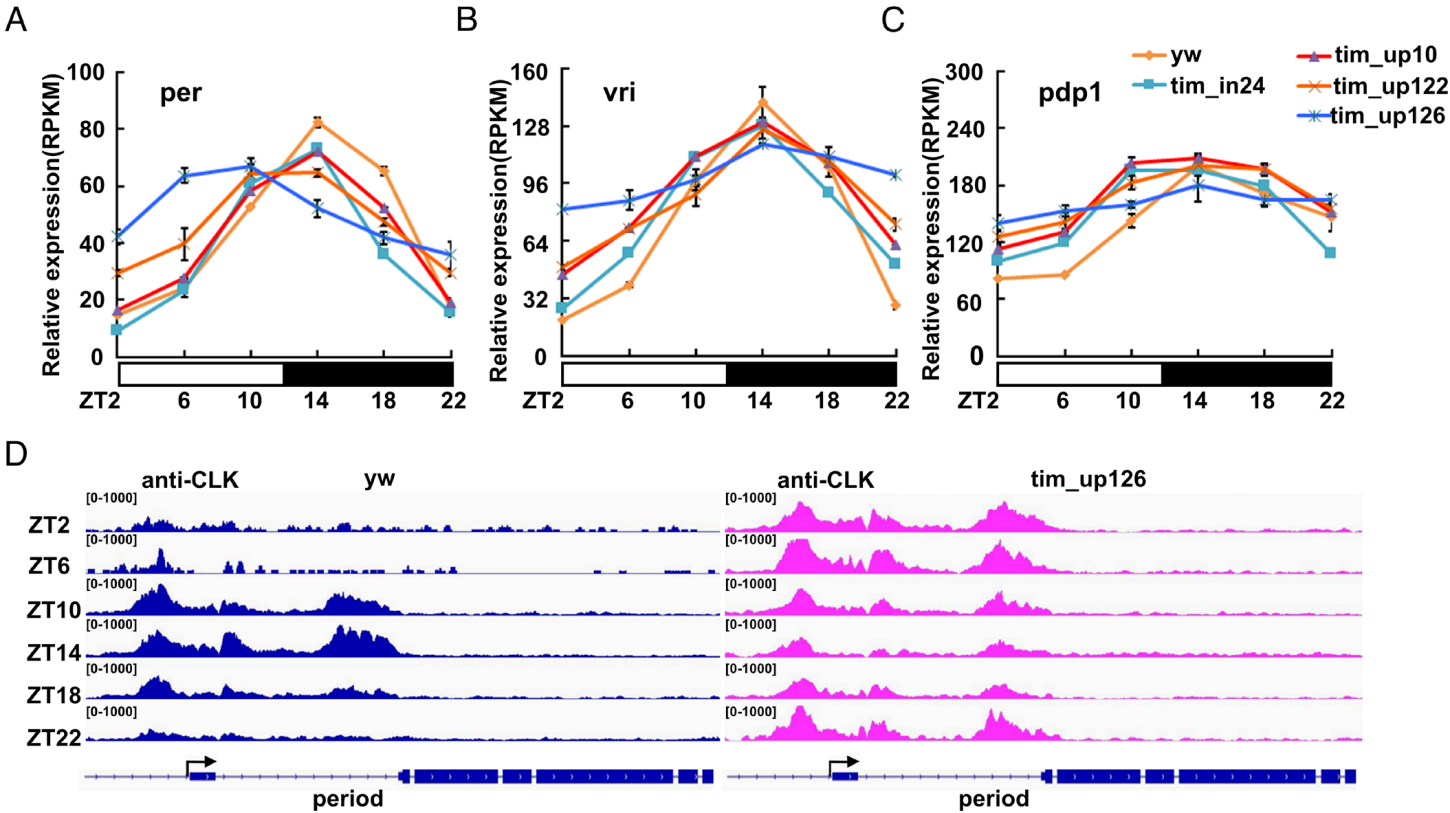
TIM represses clock gene transcription by affecting CLK DNA binding. (*A*–*C*) RNA sequencing was performed on both mutant and wild-type (*yw*) heads collected at six time points throughout the day. mRNA levels of *per* (*A*), *vri* (*B*) and *pdp1* (*C*) at each time point were quantified and graphed to show the changes that occur throughout the day. For *tim_up122*, *tim_up126*, error bars represent the SD of two biological replicates. The *y*-axis shows RPKM values normalized to the maximal level of mRNA during the day. (*D*) ChIP results show CLK binding at the *per* locus in wild-type (*yw)* (*Left*; blue) and *tim_up126* (*Right*; pink) across six time points throughout the day. In *tim_up126*, CLK binding to the *per* locus is shifted to earlier in the day.

Although there is a prominent effect of the deletions on mRNA cycling amplitude, this is largely due to a failure to achieve the low mRNA trough levels characteristic of the WT strain (Fig. 2 *A*–*C*). This is best seen for the *vri* graph (Fig. 2*B*): the allelic series has progressively increasing trough levels, and the largest deletion *tim_up126* has the highest trough levels and the lowest cycling amplitude (blue). The two other genes shown, *per* and *pdp1*, have a comparably low cycling amplitude in the largest *tim_up126* strain, also due principally to high trough values (Fig. 2 *A* and *C*). The data overall indicate an important contribution of TIM to transcriptional repression, i.e., the lower TIM levels compromise repression.

These data (Fig. 2 *A*–*C*) have a number of other features that are worth noting. 1) Consistent with the marginal effect of the *tim_in24* deletion on *tim* gene expression peak levels (Fig. 1*C*), there is almost no effect of this deletion on trough levels of the different mRNAs. 2) There is only a modest effect of most of the deletions on peak mRNA levels. 3) *pdp1* mRNA levels are almost unchanged as a function of time in the *tim_up126* deletion strain, i.e., they are strongly affected with a cycling amplitude at or near-zero. 4) There is an interesting advanced phase effect on *per* RNA cycling in the *tim_up126* strain. As this effect is matched by the advanced phase of the Clk ChIP profile, it suggests that this is a transcriptional effect due perhaps to the markedly lower levels of the PER–TIM repressor (Fig. 2 *A* and *D*). However, other CLK-CYC direct target genes are not so strikingly advanced, further suggesting a somewhat different regulation of *per* transcription.

Less robust transcriptional repression of CLK-CYC direct target genes might involve less robust release of CLK-CYC from chromatin at times when the transcription rate of these genes is relatively low (32). To address this possibility, we assayed by ChIP-seq (Chromatin-immunoprecipation followed by sequencing) the association of CLK with clock gene regulatory regions in the *tim_up126* mutant strain as a function of time of day. We assayed in parallel a WT strain as a positive control, as this result has been previously reported (23, 32). *per* and *pdp1* genes are shown as their CLK binding pattern is approximately characteristic of all well-known direct target genes (Fig. 2*D* and *SI Appendix*, Fig. S2).

CLK:CYC manifests a canonical association pattern with the *per* regulatory regions in a wild-type (*yw*) strain, namely, binding is maximal at ZT10 and ZT14 and very low at the beginning and end of the day, similar to *per* transcription and RNA profiles (Fig. 2 *D*, *Left*). The pattern is very different in the *tim_up126* strain (Fig. 2 *D*, *Right*), suggesting that the usual temporal association pattern of CLK:CYC with its direct target genes—especially the weak binding normally observed at the beginning and the end of the day when transcription is low—is affected by a lack of sufficient TIM. Although the patterns are not completely identical on all direct target genes this feature is consistent.

It is worth noting in this context that although the *tim_up126* deletion accidentally created a canonical E-box (Fig. 1*B*), the phenotype of this deletion is more severe than that of the smaller *tim_up122* deletion, which does not create an E-box (Fig. 2 *A*–*C*). This suggests that a simple E-box is insufficient to recruit normal CLK/CYC activity in this in vivo context.

### Circadian Neuron Clock Gene Expression Is Still Robust In the *tim_up126* Strain.

Fly head extracts are a traditional substrate for *Drosophila* circadian biochemistry and encompass a heterogeneous mix of tissues, including photoreceptors, fat body, neurons, and glia. Indeed, previous studies report that different circadian tissues exhibit highly heterogeneous sets of molecular clock outputs: there are only 14 common cycling transcripts between brain, fat body, gut, and malphigian tubules. Because eight of them are transcribed from the core clock genes *tim*, *vri*, *per*, *cry*, *Clk*, *cwo*, *Pdp1*, and *Cipc* (33), the core circadian oscillator is likely much more similar between tissues and cell types than their output transcripts. Nonetheless, core clock components are subject to heterogeneous splicing in an environment- and cell type–specific fashion (34–37), suggesting that there may be other differences in the regulation of core clock component between different head tissues. We were particularly interested whether the detailed regulation of *tim* transcription within the 150 clock neurons of the fly head is different from that observed in head extracts (Figs. 1 and 2). As mentioned above, this is because clock neurons dictate circadian locomotor activity rhythms and because their biochemical properties are likely invisible in fly head extracts (38).

**Fig. 3.**
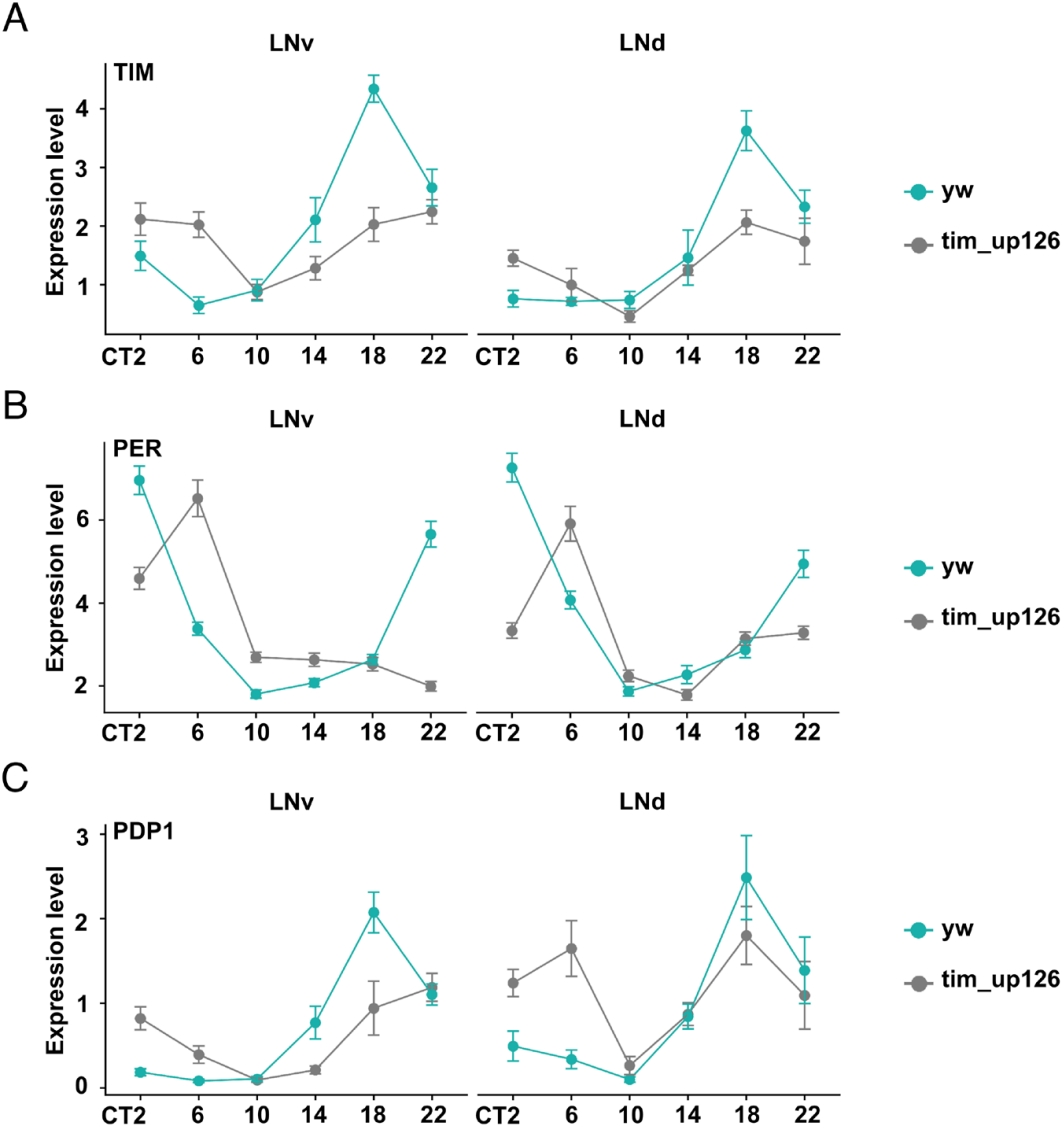
Immunostaining for TIM, PER, and PDP1 reveals that the components of the molecular clock are still properly expressed in the pacemaker neurons of the *tim_up126* mutant. (*A*–*C*) Quantification of the immunostaining signals of TIM (*A*), PER (*B*), and PDP1 (*C*) in *yw* (blue line) and *tim_up126* (gray line). Flies were entrained in LD condition for 4 d before being subjected to constant darkness condition for 3 d. Brains were dissected for immunostaining at time points throughout the third day in constant darkness. Error bars represent the SEM.

We first carried out TIM immunostaining around the clock in the *tim_up126* strain in constant darkness conditions, i.e., the same conditions under which circadian behavior is normally assayed. Although the TIM expression phase is somewhat shifted and TIM levels somewhat lower in the deletion mutant strain than in the *yw* strain, (Fig. 3*A*), the curves are much more similar than the dramatically different TIM western blot results from head extracts (Fig. 1 *D* and *E*). PER immunostaining of the *tim_up126* strain was similarly shifted compared to *yw* (Fig. 3*B*), which is very different from the PER western blot results from head extracts (*SI Appendix*, Fig. S3). Moreover, PDP1 was still cycling in clock neurons of the *tim_up126* strain albeit with lower peak levels (Fig. 3*C*). This is also very different than *pdp1* mRNA cycling in heads; levels are high and virtually noncycling (Fig. 2*C*). The staining results indicate that TIM expression and the molecular clock more generally function quite well in the clock neurons despite the *tim_up126* deletion.

The much stronger TIM expression pattern in the clock neurons could be due to altered transcriptional regulation in these cells or to some compensating regulation like enhanced protein stability. To distinguish between these possibilities, we used the single molecule in situ hybridization platform RNAScope to assay the effect of the *tim_up126* deletion on *tim* mRNA levels in the clock neurons and in glia (39). We also considered that the effects of the *tim_in24* deletion could be informative and therefore assayed this strain in parallel (40).

**Fig. 4.**
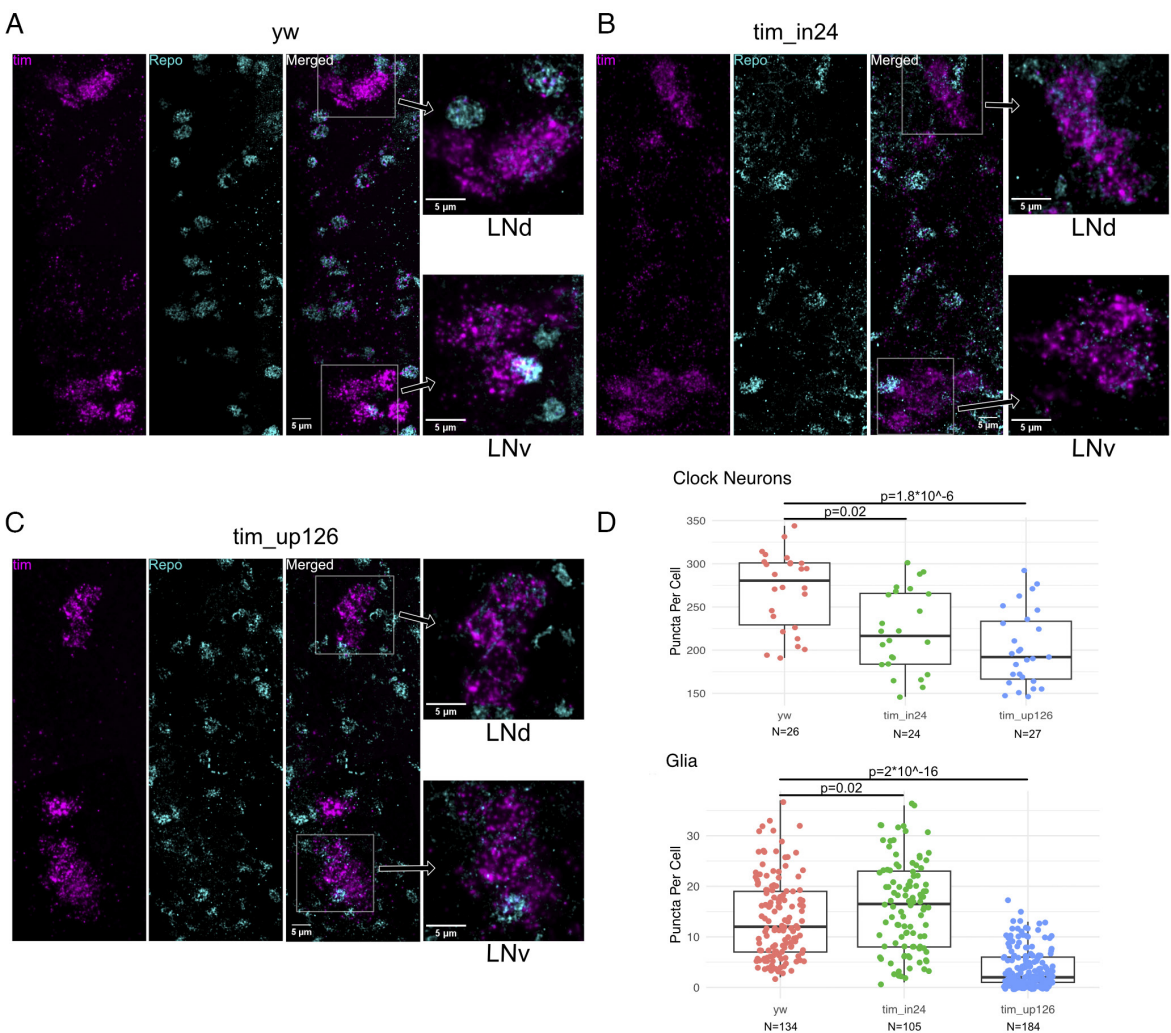
The pacemaker neurons *in tim_in24* and *tim_up126* exhibit modestly reduced *tim*, while *tim_up126* glia exhibit severely reduced *tim*. (*A*–*C*) Fluorescence in situ hybridization with RNAScope was performed to detect expression of *tim* mRNA in *yw* (*A*), *tim_in24* (*B*), and *tim_up126* (*C*) brains at ZT16. One representative max-Z projected brain hemisphere is shown per condition, along with representative Z-projected slices with no overlapping cells, demonstrating the punctate signal in the dorsolateral neurons (LNds) and ventrolateral neurons (LNvs) used for quantification in *D*. (*D*) Puncta quantification in Clock neurons (*Top*) and glia (*Bottom*) across conditions (four brains per genotype, two replicates with two brains each). Glia are defined by Repo signal, while Clock neurons are defined as cells outlined by *tim* signal with no repo signal. Statistical analysis was conducted with a one-way ANOVA with the post hoc Tukey HSD test.

The in situ hybridization data parallel what was observed by immunohistochemistry (Fig. 3), namely, a modest effect of the *tim_up126* deletion on *tim* mRNA levels in clock neurons and a strong effect in glia (Fig. 4). The modest effect of this upstream deletion is mirrored by a similarly modest effect of the *tim_in24* deletion on clock neuron *tim* mRNA levels. In contrast, there is little or no effect of the intronic deletion on *tim* mRNA levels in glia, the opposite of the strong *tim_up126* deletion effect in glia. We conclude that *tim* expression in glia relies predominantly if not exclusively on the 126 upstream enhancer region, whereas enhancer activity responsible for *tim* expression in clock neurons is shared between this region and the intronic region.

### Clock Neurons But Not Glia Utilize a Much More Active *tim* Intron Regulatory Element.

**Fig. 5.**
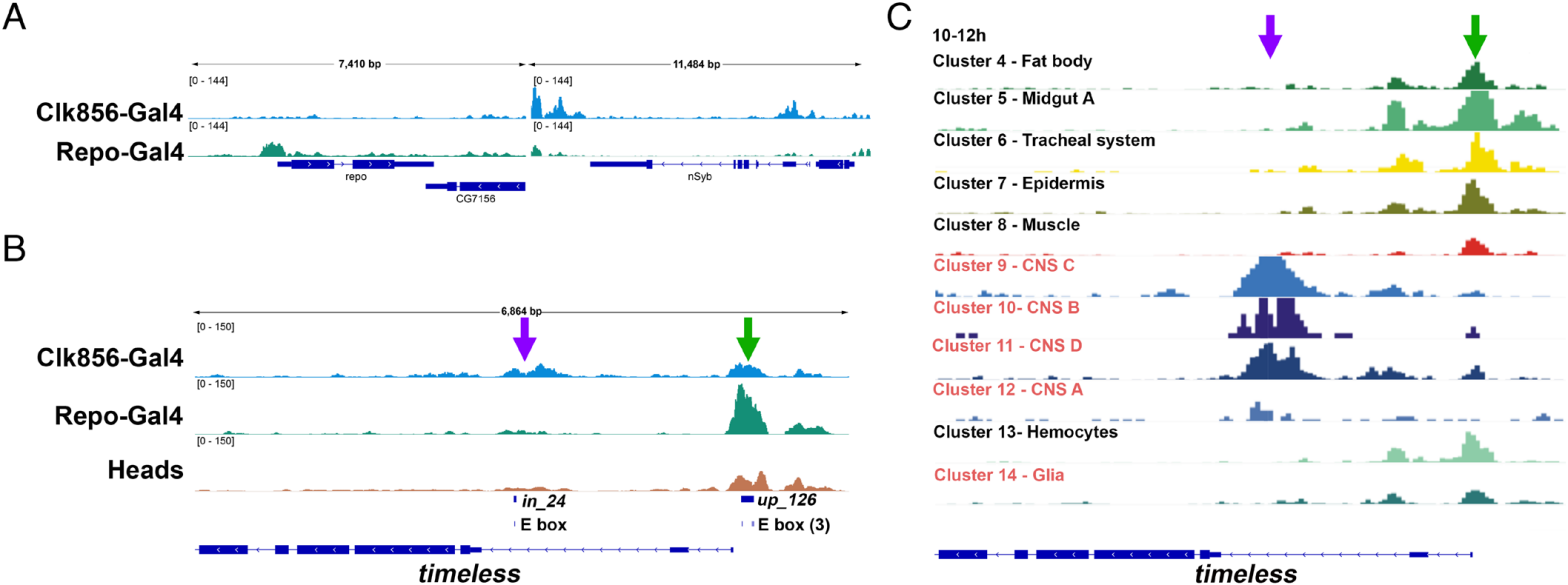
Distinct chromatin accessibilities in the intronic and upstream E-Box regions of *tim* genome. (*A*) The results of ATAC-Seq analysis from glia (green) and clock neurons (blue) are shown for glial and neural marker genes—repo and nSyb. (*B*) The chromatin accessibilities of the *tim* regulatory region in glia, clock neurons and heads. Each track is consistent with three other replicates performed for the same cell type. Flies were entrained in LD condition at least for 3 d before the experiments. Approximately 5,000 cells for glia and clock neurons were collected by Fluorescence-activated cell sorting (FACS). For ATAC-Seq from heads was done using 10 heads per replicate. (*C*) The *tim* intronic E-box region has accessible chromatin in neural cells but not in glia since an early developmental stage (10 to 12 h after egg laying). Figure *C* was directly taken from the single-cell ATAC-Seq data published on http://shiny.furlonglab.embl.de/scATACseqBrowser/. Green and purple arrows for all figures indicate the tim126 peak and intronic peak respectively.

To address this clock neuron vs. glia *tim* enhancer distinction, we used ATAC-seq to characterize gene regulatory regions from purified cells. We began by examining the nSyb and Repo genes in clock neurons and in glia. Consistent with expectation, the nSyb gene has open chromatin only in clock neurons whereas the Repo gene has open chromatin only in glia (Fig. 5*A*).

We then focused on the *tim* regulatory region and how it differs in these same two cell types; we also assayed head chromatin (Fig. 5*B*). There are two broad accessible regions in all cases, one of which corresponds to the intronic CLK binding site (purple arrow) and the other to the 126 CLK binding site (green arrow). In the case of the head pattern (Fig. 5*B*), the relative size of these two regions (126 ≫ intron) resembles their relative signal in the head CLK ChIP assay (Fig. 1*A*). This indicates that the head ATAC-seq pattern simply reflects the much greater CLK binding to the 126 region than to the intronic region in heads and suggests that a similar CLK binding ratio occurs in glia. In fact, Repo-Gal4 cells have even less relative intronic region signal than heads (126 ≫≫ intron; Fig. 5*B*), suggesting that there is even less relative CLK binding to the intronic region of glia, perhaps because heads are comprised of mixed cell types—some of which may have more intronic CLK binding. The absence of any substantial intronic ATAC-seq signal provides an explanation for why there is little if any glial circadian gene expression without the upstream 126 region.

Clock neurons (Clk856-GAL4 cells) in contrast are very different: They have much more ATAC-seq intronic signal, comparable to the signal found at their 126 region (Fig. 5 *B*, *Top*). This provides a mechanistic explanation for why circadian neuron *tim* gene expression is substantial despite the 126 deletion: The intronic region alone is sufficient to recruit sufficient CLK in circadian neurons but not in glia (Fig. 5; *Discussion*).

### What Might Determine These Cell Type Differences between ATAC-Seq Patterns?

We considered that there might be a developmental factor that opens or reflects a greater opening of the *tim* intronic E-box region of chromatin in neurons relative to glia. Consistent with this prediction is a striking open chromatin region over the intronic E-box in neurons but not in glia; this open region is present in published ATAC-seq patterns during fly embryonic development (Fig. 5*C*). As this region appears before the appearance of *Clk* gene expression (41), these temporal data suggest that another factor opens the chromatin at the *tim* intronic E-box region during embryonic development, which ultimately enables its subsequent interaction with much more CLK in circadian neurons than in glia.

Alternatively, a quantitative difference in CLK expression between adult clock neurons and glia might exist and contribute to the distinction in ATAC-seq patterns between these cell types. To address this second possibility, we overexpressed CLK in glia with UAS-CLK and Repo-Gal4. Unfortunately, only adult overexpression was possible. This was enabled by the auxin-inducible gene expression system (AGES) and was necessary because the simpler strategy of UAS-CLK expression with Repo-GAL4 throughout development was lethal; this was also the case with nSyb-GAL4; see below. Nonetheless, we considered that adult overexpression might still be effective because mammalian CLK has been shown to act as a pioneer transcription factor by opening closed chromatin (42).

**Fig. 6.**
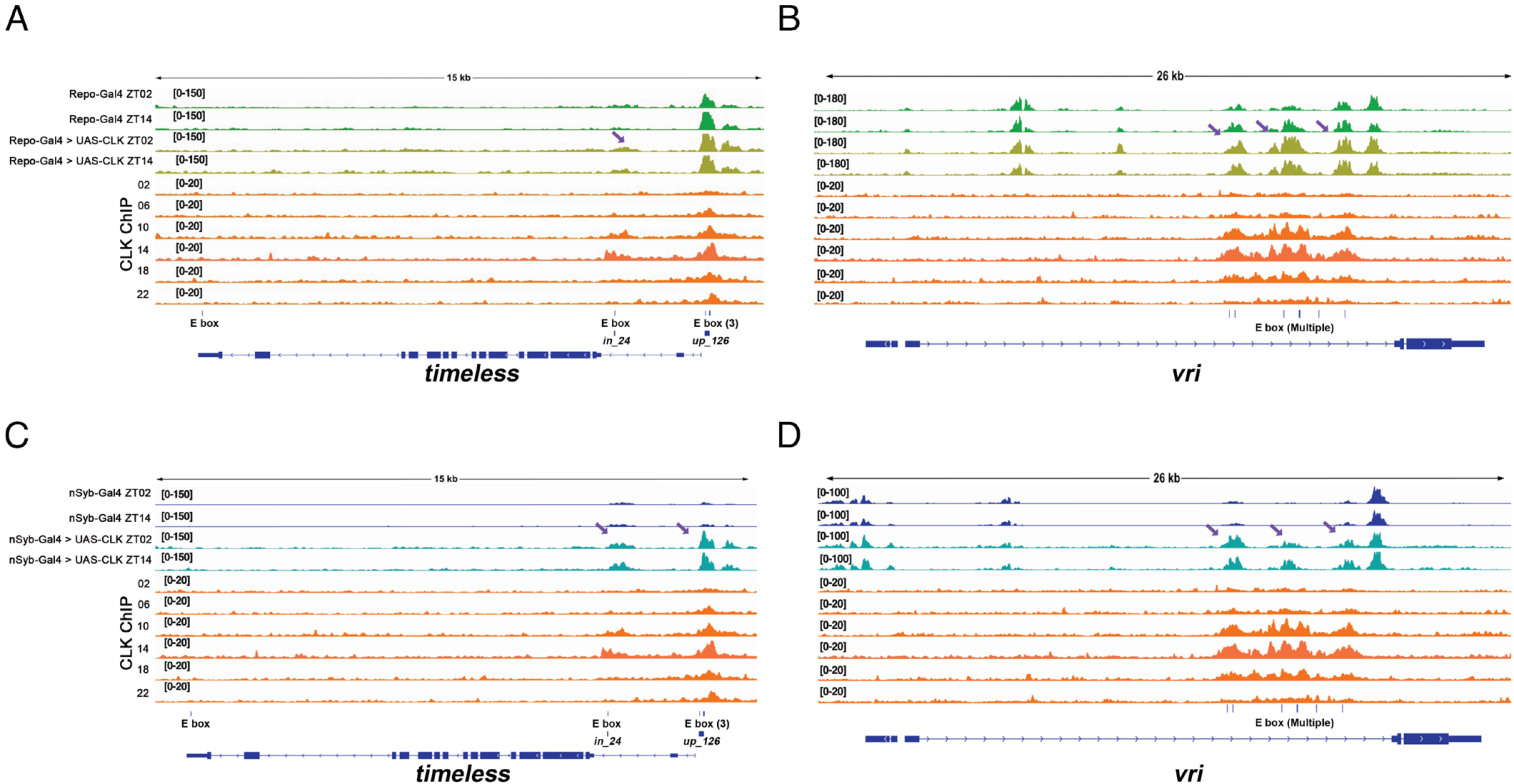
CLK facilitates the accessibility of core clock gene regulatory regions. (*A*–*D*) UAS-CLK flies were crossed with Repo-GAL4; AGES-Gal80 and nSyb-Gal4; AGES-Gal80 to achieve adult-specific CLK expression. Two-week-old progeny were fed 10 mM auxin for 5 d while being entrained in LD condition. Flies were collected at ZT02 and ZT14 and 5,000 cells per replicate were used for tagmentation. ATAC-Seq tracks are aligned with tracks for CLK-ChIP (around the clock). (*A* and *B*) Adult-specific overexpression of CLK in glia results in a modest yet noticeable increment in chromatin accessibility within E-box regions of *tim* (*A*) and *vri* (*B*). (*C* and *D*) Adult-specific expression of CLK in neurons results in dramatically enhanced chromatin accessibility of the *vri* (*C*) and *tim* (*D*) genes. Both neural and glial-specific overexpression of CLK have at least two replicates. Purple arrows indicate regions of increased chromatin accessibility driven by CLK overexpression.

The results indeed indicate that adult CLK overexpression in glia increased the ATAC-seq signal within the intronic E-box region of the *tim* gene (Fig. 6 *A*, *Left*, purple arrow; compare the change of these peaks to the no change in the flanking control peaks). The effect however is modest. Moreover, there is a comparable and perhaps even greater effect on the upstream 126 E-box region, indicating that the dramatic ratio of open chromatin between the two regions (126 ≫ intronic) is not appreciably altered. There is a similar modest positive effect on the ATAC-seq signals corresponding to the CLK-binding peaks within the *vri* gene regulatory region (Fig. 6*B* and *Discussion*), suggesting that the increase in CLK-binding peak signal is general. Although caveats exist (*Discussion*), this argues against a simple version of this second possibility.

To provide a more general context to the effect of adult CLK overexpression, we expressed UAS-CLK in most if not all adult neurons with nSyb-GAL4 as well as the AGES system (Fig. 6 *C* and *D*). The results were qualitatively similar to Repo-gal4 CLK adult overexpression, e.g., an enhanced ATAC-seq signal in the CLK-binding region within the *vri* gene (Fig. 6*B*). However, neuron overexpression resulted in a much larger signal increase in this region of the *vri* gene (Fig. 6*C*, indicated by purple arrows). CLK overexpression causes an even more dramatic signal increase in the relevant regions of the *tim* gene (Fig. 6*C*).

We interpret the dramatic quantitative difference between glia and neurons to the fraction of these cell populations that contain a molecular clock in wild-type fly heads; many, most, and perhaps all glia contain a molecular clock, whereas there are only 150 circadian neurons in the adult fly brain. Although this number is likely to be an underestimate of the number of neurons with a molecular clock, it is likely that most fly brain neurons do not contain a robust molecular clock. Consistent with the notion that the control nSyb ATAC-seq signal reflects the 150 neurons, the ratio of the two *tim* regions is approximately equal (Fig. 6*C*), similar to its ratio in clock neurons. We therefore suggest that the very large increase in ATAC-seq signal with nSyb-GAL4 represents the de novo recruitment of naïve adult brain neurons to become circadian neurons. This notion is based on the previously observed effect of ectopic CLK expression in flies (43, 44) as well as its chromatin remodeling properties in mammals (42).

### *tim* Deletion Effects on Behavioral Rhythmicity.

**Fig. 7.**
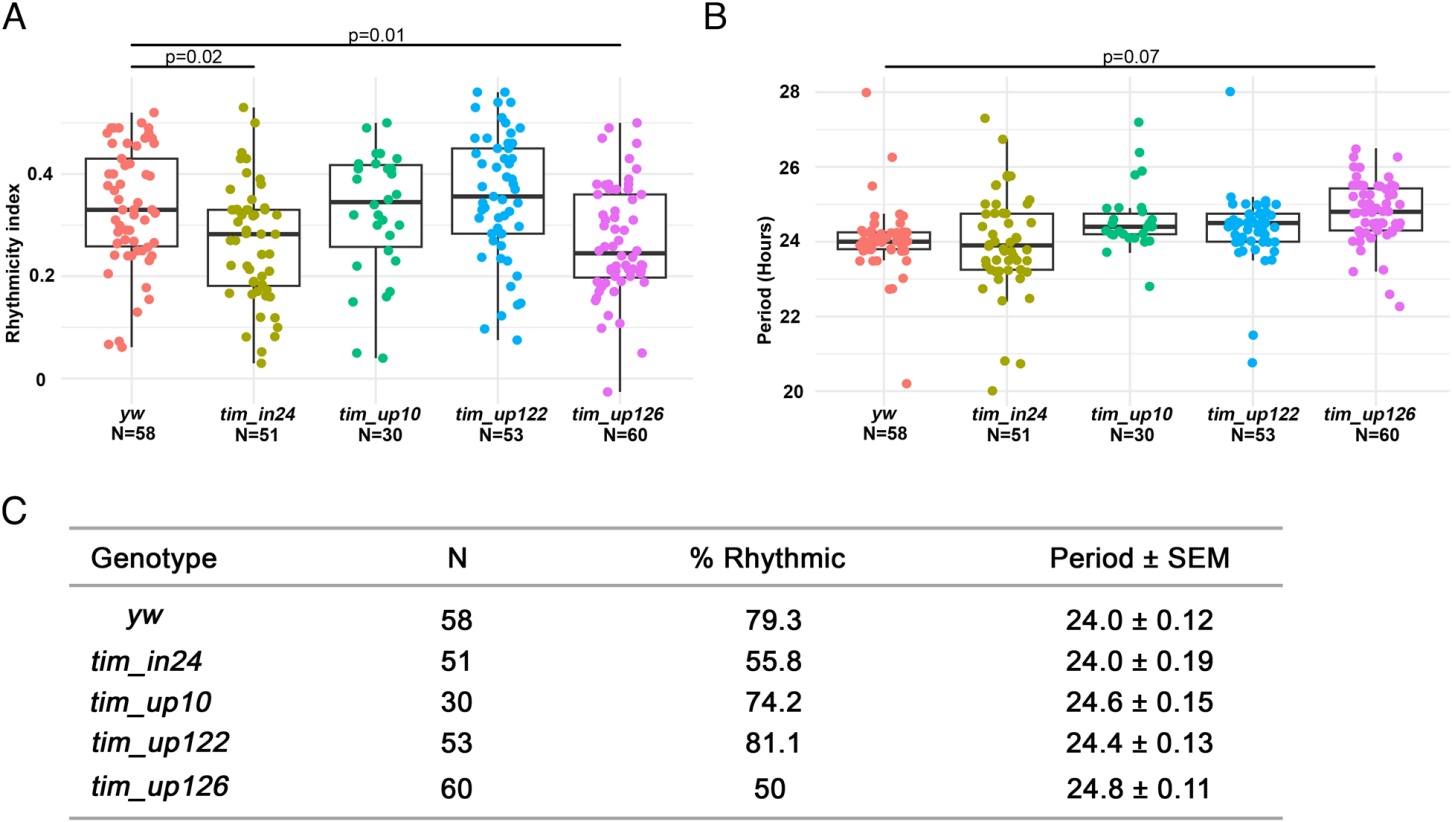
Deletions in the promoter and intronic sequence of *tim* affect rhythmicity and period. (*A* and *B*) Quantification of rhythmic index (*A*) and period length (*B*) of the E-box deletion mutants in free running conditions. (*C*) Table of rhythmic features calculated from *A* and *B*. Flies with a rhythmic index greater than 0.25 were defined as rhythmic. Male flies were entrained in LD conditions before being subjected to constant darkness condition for 7 d at 25 °C. Flies from two replicate experiments were analyzed together. Statistical analysis was conducted with a one-way ANOVA with the post hoc Tukey HSD test.

The *tim* expression and clock gene cycling results (Figs. 3 and 4) as well as the ATAC-seq data (Fig. 5) beg the question, what are the deletion effects on circadian behavior? Not surprisingly perhaps, the key deletion strains have comparable decreases in rhythmicity, most notably the *tim_up126* (*P* = 0.007) and *tim_in24* (*P* = 0.01) strains (Fig. 7*A*), which is consistent with the comparable ATAC-seq signals on these two regions in clock neurons. This suggests that elimination of either enhancer reduces *tim* expression to approximately the same extent (Fig. 4), with a comparable effect on the rhythmic index. Although the data do not allow for a more quantitative assessment, it is interesting that a likely modest decrease in *tim* expression and molecular cycling amplitude within clock neurons affects rhythmic index rather than period length (*Discussion*).

The deletion effects on the circadian period are different from those on the rhythmic index. The upstream deletion strains have a slightly (0.5 h) longer period than WT with no statistically significant difference between them, whereas the *tim_*in24 strain is indistinguishable from WT (Fig. 7 *B* and *C*). We speculate that this distinction between the two regions may reflect a modest effect of the glial clock on the circadian period.

## Discussion

To address the contribution of canonical *cis*-regulatory elements to clock gene expression and to the *Drosophila* circadian behavioral program, we generated a series of deletions that encompass *tim* transcriptional regulatory DNA. There was a major effect of the largest upstream deletions on *tim* gene expression in heads and glia. In contrast, these deletions only had a modest effect on *tim* gene expression in clock neurons. Addressing this discrepancy let us to identify a substantial difference between glia and neurons in the extent of open chromatin at the intronic E-box region. The findings have implications for the tissue-specific regulation of circadian gene expression and extend to more general considerations of transcription factor activity as well as to interpretations of quantitative differences in ATAC-seq signals between tissues. Most data in the literature address qualitative ATAC-seq differences between tissues.

The deletion strains were made with CRISPR and effectively constitute an allelic series that progressively decrease *tim* gene expression in heads; the *tim_up126* strain has the biggest deletion and reduces the amplitude of *tim* RNA cycling and peak levels to about 15% of normal with similar effects on TIM protein levels. Although the cycling amplitude of other clock genes is also reduced, these effects are very different from those on *tim*; they increase trough levels rather than reduce peak levels (Fig. 2 *A*–*C*). Moreover, there is much more robust CLK/CYC chromatin binding in the *tim_up126* deletion mutant strain at times when transcription and chromatin binding are normally low in the WT strain, i.e., ZT2, ZT6, and ZT22 (Fig. 2*D*). Decreased TIM expression levels result in a lower amount of PER–TIM complex within some or all clock neurons. This leads to incomplete repression of CLK/CYC binding activity, contributing to reduced cycling amplitudes of clock mRNAs like *per*, *vri*, and *pdp1*. These data suggest that TIM like PER contributes directly to transcriptional repression via removal of CLK/CYC from chromatin, i.e., the low overall TIM levels in the *tim_up126* strain enhance clock gene expression at these times. Indeed, TIM and PER probably ChIP identically to chromatin, suggesting that a PER–TIM dimer is the on-chromatin transcriptional repressor, analogous to the mammalian PER-CRY repressor.

All of these considerations apply to fly heads, which contain a complex mix of cell types. The eyes and glia are known to contain robust molecular clocks and dominate traditional circadian biochemistry and gene expression results from heads. Signals from these two tissues and perhaps other head cell types likely dwarf the signals from the 150 clock neurons, which are then probably invisible in head data. Importantly, the *tim_up126* deletion effect on clock neuron *tim* expression is much less severe than its effect on head and glial *tim* expression (Figs. 3 and 4). This distinction is well illustrated by the modest effect of the *tim_up126* deletion on *tim* gene expression in at least some and perhaps all circadian neurons as well as by comparing its effect on the *tim* RNA signal between the clock neurons and glia (Fig. 4). Intriguingly, the *tim_in24* deletion has a modest effect on clock neuron *tim* expression approximately equal to that of the *tim_up126* deletion but no effect on glial *tim* expression.

What accounts for these deletion effects? The larger upstream deletions remove the CLK-binding region, which contains three E-boxes and is the major CLK binding site in heads; only the minor single E-box–containing intronic enhancer remains. ATAC-seq profiles as well as CLK ChIP assays indicate that CLK binds poorly to this intronic region in heads and in glia as expected. Although we have not done cell-specific CLK ChIP assays, we suspect that the much more prominent ATAC-seq profile of this region in circadian neurons (Fig. 5) likely reflects its stronger CLK binding as well as a more shared distribution of CLK binding between the two regions in these neurons.

These ATAC-seq data can also explain why the large upstream deletions only have a modest effect on circadian behavior. Indeed, their impact on rhythm strength is about the same as the impact of the intronic enhancer deletion (Fig. 7), reflecting the roughly similar enhancer activities of the two regions (Fig. 5). These features indicate a specialized, cell-specific role for the intronic enhancer and are reminiscent of the “shadow enhancer” model of gene regulation (45, 46). This model suggests that the intronic enhancer is necessary for robust *tim* expression despite environmental challenge, a possibility we are keen to explore. Perhaps other shadow enhancers will be similarly featured by additional phenotyping or more detailed characterization.

Although it is not surprising that reduced cycling amplitude of clock gene expression affects rhythm strength, there is not much precedence for this relationship in the literature. There is in addition a small period-lengthening effect of only the upstream deletions. It is tempting to consider that this is an effect of dramatically reduced *tim* expression and clock gene cycling only in glia, a possibility we will test.

What is the explanation for the substantially more open intronic ATAC-seq pattern in clock neurons compared to glia? A published ATAC-seq analysis indicates that a factor binds to this region of *tim* regulatory chromatin in neurons but not in glia during embryonic development (Fig. 5). This factor may license more CLK binding at this location before disappearing later in development. A second possibility is a relevant adult neuron-specific factor. It could open chromatin of the *tim* intronic region and/or even bind to this region, perhaps adjacent to CLK. Another possibility is that neurons have more CLK or higher CLK activity than glia, promoting more CLK binding to the less efficient intronic E-box. We tried to address this last possibility by overexpressing CLK in glia. The results were negative, namely, the skewed glial ATAC-seq pattern was not converted into the more symmetric neuronal pattern (Fig. 6). This was perhaps because we could not overexpress CLK during development due to the lethality of overexpression. There was however a modest increase in the ATAC-seq signal of both regions, suggesting that signal intensity may parallel an increase in in vivo CLK activity. A simple mechanistic interpretation is that increased CLK levels decrease the CLK off-rate, which might be inversely related to ATAC-seq signal strength.

However, a different explanation is required for the much larger increase in ATAC-seq signals by overexpressing CLK in most or all adult neurons with nSyb-GAL4 (Fig. 6). We suggest that these dramatic increases reflect CLK activity in many neurons that normally never express CLK. The result recalls the ability of CLK expression to effect ectopic brain clocks (43, 44). These previous studies expressed CLK throughout development, but our current result suggests that adult expression can also create ectopic clocks; a further suggestion is that even adult CLK expression can effectively open chromatin (42).

Although there is at present no definitive mechanistic explanation for the ATAC-seq pattern differences between neurons and glia, there is an attractive teleological explanation. Although each cell has only one copy of *tim* regulatory DNA, the cell size and RNA content of circadian brain cells differ substantially, e.g., glia may be on average smaller cells than clock neurons. The larger cells may require more gene expression from each genome to synthesize more clock gene RNA and protein per cell. Because the clock molecules are degraded every 24 h, a greater rate of synthesis may be the only way to achieve higher peak levels in larger cells. Although higher synthesis rates can occur in a number of ways, recruiting more CLK via the intronic regulatory region as well as the 126 region in the case of neurons can enable more robust clock gene transcription compared to the 126 region alone in glia.

The difference between CLK binding to these two *tim* regulatory regions recalls mammalian data indicating that multiple, adjacent E-boxes rather than single E-boxes are preferred in clock gene regulatory DNA (15). This preference is underscored in recent structural work characterizing CLK:BMAL1 binding to a pair of E-boxes in the presence of histones (47). The work here indicates that a similar preference of CLK-CYC binding to multiple E-boxes may exist in flies, but the quantitative differences between tissues suggest that somewhat different rules may govern binding within neurons. It will be interesting to see whether a similar distinction emerges from future work in mammals comparing CLK-BMAL1 binding between the liver and SCN.

## Methods

### *Drosophila* Lines.

Flies were housed in standard cornmeal/agar medium with yeast under 12:12 h LD (light:dark) cycles*. yw* flies were used as the wild-type control. Flies used in this study are in *SI Appendix*, Table S1. CRISPR lines were generated by injecting pCFD3-dU6:3gRNA (Addgene, #49410) or pCFD4-dU6:3tandemgRNAs (Addgene, #49411) containing guide RNAs (gRNAs) corresponding to either the *tim* promoter or the intronic E-box sequences into embryos using Rainbow Transgenic (Cambridge, MA). *tim_in24*, *tim_up10* were generated by a single gRNA. *tim_up122, tim_up126* were generated by tandem gRNAs, the primers used to generate the mutants are listed in *SI Appendix*, Table S2. The CRISPR-generated deletions were validated by sequencing the region. Fly lines homozygous for the CRISPR-generated *tim* deletions were used for all subsequent biochemical and behavioral analyses.

### RNA Extraction, qRT-PCR and Next Generation Sequencing.

Male and female flies were entrained in 12:12 LD conditions for 4 d at 25 °C, and then collected at ZT2, ZT6, ZT10, ZT14, ZT18, and ZT22. Total RNA from fly heads was extracted using TRIzol reagent (Invitrogen) following the supplier’s protocol. The resulting RNA was used to make libraries for transcriptome sequencing using the TrueSeq RNA Sample Prep Kit (v2; Illumina). The quality of the libraries was assessed using a Bioanalyzer 2100 (Agilent) and then sequenced on a NextSeq 550 (Illumina). RNA libraries were mapped to *Drosophila* genome dm6 using Tophat with default setting and expression levels were quantified using ESAT (48, 49). For qRT-PCR, the extracted RNA was quantified by Nanodrop (Thermal Fisher), 500 ng of total RNA was used to synthesize cDNA with a TAKARA kit following the standard protocol. The cDNA was diluted in water and used as a template for quantitative PCR using the qPCRBIO SyGreen Blue Mix (Genesee Scientific). *Rpl32* served as an internal control.

### Western Blots.

Wild-type and *tim_up126* mutant flies were entrained in 12:12 LD condition for 4 d at 25 °C, and then collected on dry ice every 4 h throughout the day. Fifty fly heads were homogenized in RIPA buffer with protease inhibitors (Roche) and phosphatase inhibitors (Fisher). Protein extract was heat-denatured in SDS buffer (200 Mm Tris-HCl pH 6.8, 8% Sodium Dodecyl Sulfate (SDS), 0.4% bromophenol blue, 40% glycerol) with 100 °C for 5 min. The samples were separated on a 3 to 8% Tris-acetate gel (Invitrogen) and transferred to nitrocellulose (iBlot; Invitrogen). Blots were blocked in 5% milk in PBST (3.2 mM Na2 HPO4, 0.5 mM KH2PO4, 1.3 mM KCl, 135 mM NaCl, and 0.05% Tween-20, pH 7.4) for 1 h and incubated overnight with either rat anti-TIM at 1:4,000 dilution, rabbit anti-PER at 1:4,000 dilution or mouse anti-Actin (loading control; Santa Cruz) antibodies. Western blot intensity was quantified using Image J.

### ChIP-Seq.

ChIP-seq was performed on *yw;; WT dCLK-V5* (wt) and yw; *tim_up126; dCLK-V5* as previously described with the following exceptions (50). Three- to five-day-old flies were entrained in LD condition for at least 3 d and then collected every 4 h throughout the day. Fly heads were homogenized in homogenization buffer (10 mM HEPES-KOH (4-(2-Hydroxyethyl)piperazine-1-ethanesulfonic acid) at pH 7.5, 10 mM KCl, 1.5 mM MgCl2, 0.8 M sucrose, 0.5 mM EDTA (Ethylenediaminetetraacetic acid), 1 mM DTT (1,4-Dithiothreitol), 1× protease inhibitor, 1× phosphatase inhibitor cocktail) at 4 °C. Homogenates were loaded on equal volumes of sucrose cushion buffer (with 1.0 M sucrose and 10% glycerol in the homogenization buffer) and centrifuged in a HB-6 rotor (Sorvall) at 11,000 rpm for 10 min at 4 °C. Nuclear pellets were suspended in 1 mL 1× PBS with 1% formaldehyde and fixed for 15 min at room temperature and quenched by 0.125 M glycine. Chromatin was extensively washed by 1× PBS and sheared using a biorupter (Diagenode) in 500 µL sonication buffer (20 mM Tris-HCl at pH 7.5, 150 mM NaCl, 10% glycerol, 1% SDS). For each IP, 25 µL of sheared chromatin was reserved as input sample. The remaining chromatin sample were diluted by 10 times of IP buffer (20 mM Tris-HCl at pH 7.5, 150 mM NaCl, 10% glycerol, 1% NP40 and protease inhibitor tablet) and incubated with 30 µL anti-V5 agarose beads (Sigma) overnight at 4 °C. Beads were washed and eluted and decrosslinked as described previously. The DNA was purified using Minelute columns (Qiagen). Of note, 30 μL of DNA from each IP or 50 ng of input DNA were used to generate ChIP-seq libraries according to the Illumina ChIP-seq protocol. After adaptor ligation, DNA samples were separated by 2% agarose TAE (Tris-acetate-EDTA) gel and gel slices corresponding to 250 to 450 bps were recovered and purified using the QIAGEN gel purification kit. ChIP-seq libraries were sequenced on a Nextseq 550 (Illumina) and the resulting datasets were mapped to the *Drosophila* genome (dm6) using Bowtie2 and analyzed using MACS2 (51, 52).

### Locomotor Activity Assay.

First, 3- to 5-d-old male flies were placed into glass tubes containing 2% agarose and 5% sucrose and then entrained for 4 d in 12 h light:12 h dark (LD) conditions, followed by 4 to 5 d in constant darkness (DD) conditions using TriKinetics (Waltham, MA) DAM system (*Drosophila* Activity Monitors). Analyses were performed with MATLAB as described (53). A rhythmicity threshold of 0.3 was applied.

### Immunocytochemistry.

Immunostaining experiments were performed on 3- to 5-d-old male and female flies as previously described (54). Flies were entrained in LD conditions for 4 d before being subjected to constant darkness for 3 d. Flies were harvested for immunostaining at time points throughout the third day in constant darkness. Fly bodies were fixed in PBS with 4% (vol/vol) paraformaldehyde with 0.5% Triton X-100 for 2 h and 40 min at room temperature. Brains were dissected, washed twice in 0.5% PBST buffer and then blocked overnight in 10% Normalized Goat Serum (NGS; Jackson Immuno Research Lab) at 4 °C. The brains were then incubated in rabbit anti-PER at 1:1,000 dilution, a rat anti-TIM at 1:200 dilution or a guinea pig anti-PDP1 antibody at a 1:1,000 for 2 to 3 d (55–57). The brains were washed 3 times by PBST, then incubated with either Alexa Fluor 488-conjugated anti-rat or anti-mouse (1:1,000 dilution), and Alexa Fluor 633-conjugated anti-rabbit or anti-guinea pig at 1:500 dilutions in 10% NGS. Brains were mounted in Vectashield (Thermal Fisher) and imaged on a Leica SP5 confocal microscope, the z-stack was sequentially imaged in 1 μm sections. Image J was used for signal quantification.

### RNAScope.

Fluorescence in situ hybridization of *Drosophila* brains was performed using the RNAScope Multiplex Fluorescent Detection Kit v2 (Advanced Cell Diagnostics, ACD) using the protocol described in ref. 40 with some modifications. Flies were collected at ZT16 directly into 4% formaldehyde in PBST [PBS+ 0.5% Triton X-100] on ice and fixed for 5 h on a rotator at 4 °C in the dark. Brains were then dissected and fixed overnight in 4% formaldehyde in PBST on a rotator at 4 °C. Fixed brains were washed twice with 650 µL PBST and once with 650 µL PBST+ 1% BSA for 10 min on a rotator at room temperature (RT) before being incubated for 5.5 min in a 100 °C heat block in 550 µL prewarmed 1× Target Retrieval Solution (ACD). Brains were washed for 1 min in PBST+ 1% BSA at RT, for 1 min with 650 µL methanol at RT, then for 10 min with PBST+ 1% BSA at RT on a rotator. Brains were then postfixed with 500 µL 4% formaldehyde in PBST for 25 min on a rotator at RT and washed with PBST+ 1% BSA for 10 min on a rotator at RT. Brains were then incubated with 2 drops of Protease Plus solution (ACD) for 10 min at 40 °C. Brains were washed with 650 µL PBST+ 1% BSA for 10 min on a rotator at RT, then with 2 drops of Probe Diluent (ACD) for 1 min. Brains were incubated with 100 µL prewarmed Tim-C1 probe solution overnight at 40 °C.

Signal amplification and labeling were performed according to the manufacturer’s instructions with the following modifications. All incubations at 40 °C were performed with 2 drops of solution in a heat block, and all washing steps were done 2× for 5 min with 650 µL RNAScope Wash Buffer (ACD) on a rotator at RT. Opal 650 dye (Akoya Biosciences) was diluted 1:6,000 in TSA buffer (ACD) for conjugation to Tim-C1 probe. Brains were incubated in 300 µL diluted Opal 650 dye for 30 min at 40 °C.

For colabeling with immunohistochemistry, brains were washed 3× for 10 min on a rotator at RT with PBST after the last RNAScope Wash Buffer washes following incubation with HRP (horseradish peroxidase) Blocker Solution (ACD). Brains were then blocked in 200 µL 10% NGS on a rotator for either 2 h at RT or overnight at 4 °C. To prevent photobleaching of RNAScope signal, all longer incubations were done in the dark. Brains were then incubated with primary antibody [mouse anti-Repo, (DHSB) diluted 1:100 in 10% NGS] for 24 to 48 h at 4 °C. Primary antibody was removed and brains were washed with PBST 3× for 10 min on a rotator at RT. Brains were then incubated with secondary antibody [Alexa Fluor 488 goat-anti-mouse (Invitrogen) diluted 1:200 in 10% NGS] overnight at 4 °C on a rocker. Secondary antibody was removed and brains were washed with PBST 3× for 10 min on a rotator at RT and mounted in Vectashield (Vector Laboratories).

Images were acquired using a Leica Stellaris 8 confocal microscope with a 63× oil immersion objective. Z-stacks were acquired with 0.3 µm steps and 2,500 × 2,500 resolution.

### RNAScope Puncta Analysis.

Lightning deconvolution was performed on the tim RNAScope channel following acquisition using default settings. Images were imported into FIJI, where acquired images were exported in three separate tif files: two files with *repo* and *tim* RNAScope signal where images were adjusted to aid in segmentation, and one with unadjusted *tim* RNAScope signal for puncta quantification. Z-slices were carefully selected to avoid overlapping cells.

Segmentation was performed using Cellpose (58). For glia, segmentation was conducted on *repo* signal using the cyto model, manually fine-tuned using random Z slices across conditions. Masks produced by the fine-tuned model were subject to manual curation. For clock neurons, segmentation was performed manually, defining Clock neurons as the outline formed by high *tim* signal in regions without repo signal.

Puncta quantification was performed using FISH-quant v2 using default settings and a brightness threshold of 398 (59). Masks generated with Cellpose were used to separate signal into individual cells, with plots created for individual cells. Puncta assignments were manually verified. Statistical analysis and graphs were generated in R.

### Omni-ATAC.

ATAC-Seq was performed on FACS-sorted clock neurons and glia using Clk856-Gal4 and Repo-Gal4, crossed to UAS-unc84-GFP, respectively. Two-week-old flies were collected at ZT02 and ZT14. Overexpression of CLK in neurons and glia was conducted using a UAS-CLK strain and the AGES-Gal80 system. Adult-specific CLK overexpression was achieved by feeding flies 10 mM auxin for 5 d. For Clk856-Gal4, Repo-Gal4, and nSyb-Gal4, brains were dissected in Schneider’s media and incubated with 0.75 mg/mL Collagenase and 0.4 mg/mL Dispase at room temperature for 30 min. Brains were then triturated 50 to 80 times to obtain a single-cell suspension for FACS.

Transposition on sorted cells was performed as previously described (60). Briefly, sorted cells and nuclei were centrifuged at 1,000 rpm for 10 min at 4 °C and the pellet was resuspended in 50 µL of Transposition mix (25 µL 2× TD buffer, 16.5 µL PBS, 0.05 µL 10% v/v Tween, 0.05 μL 1% v/v Digitonin, and 2.5 µL TDE1 Enzyme (Illumina, San Diego, CA, USA Catalog #20034198) and incubated at 37 °C for 30 min. Tagmented DNA was purified using Zymo DNA clean and concentrator kit. Purified DNA fragments were amplified using single-indexed Nextera primers (Integrated DNA Technologies) for 12 PCR cycles. Amplified libraries were purified using AMPure XP beads (Beckman Coulter, Brea, CA, USA Catalog #A63880), and size distribution was accessed using TapeStation High-Sensitivity D1000 Screentape. Final libraries were sequenced on a Nextseq 550 (Illumina).

### ATAC-Seq Preprocessing.

ATAC-Seq files were adaptor-trimmed using fastp. Bowtie2 was used to aligned trimmed files using the following parameters: --local --very-sensitive-local --no-unal --no-mixed --no-discordant --phred33 -I 10 -X 700 (51). Samtools was used to remove PCR duplicates and Sambamba was used to remove multimapping reads (61). Tn5 insertion bias was corrected using a custom python script and peaks were called using MACS2 (62).

### ATAC-Seq Differential Peak Calling and Normalization.

Peaks called from MACS2 were intersected between replicates using bedtools, and peaks across conditions were merged and converted into a reference annotation in SAF format (63). Featurecounts was used to count reads in each sample within each region in the reference file, and input into DESeq2 in R. DESeq2 was used to perform differential peak quantification, and normalization factors were produced as well. Normalization factors were used to produce normalized bigwig files for visualization using Deeptools bamcoverage (64).

## Supplementary Material

Appendix 01 (PDF)

## Data Availability

The raw and processed sequencing data have been deposited in GEO under accession numbers GSE259243 (ATAC-Seq) (65), GSE259245 (RNA-Seq) (66), and GSE259247 (ChIP-Seq) (67).
