# Supplementary material for: Timeless noncoding DNA contains cell-type preferential enhancers important for proper Drosophila circadian regulation
Source: Proc Natl Acad Sci U S A. 2024 Apr 3;121(15):e2321338121. doi: 10.1073/pnas.2321338121 (PMC11009632; doi:10.1073/pnas.2321338121)
Supplement: Supplementary file 1 — Appendix 01 (PDF) [file pnas.2321338121.sapp.pdf]

PNAS

Supporting Information for

timeless non-coding DNA contains cell-type preferential enhancers important for proper Drosophila circadian regulation

Dingbang Ma, Pranav Ojha, Albert D. Yu, Maisa S. Araujo , Weifei Luo[5], Evelyn Keefer, Madelen M. Díaz, Meilin Wu, William J. Joiner, Katharine C. Abruzzi, Michael Rosbash*

*Michael Rosbash

Email: rosbash@brandeis.edu

This PDF file includes:

Figures S1 to S3:

Figure S1. qRT-PCR verification of *tim* expression in E box deletion mutants.

Figure S2. TIM affects CLK DNA binding activity to regulate *pdp1* transcription.

Figure S3. PER expression is dramatically reduced and shows no robust cycling in the head of tim_*up126* under LD conditions.

Table S1 and S2:

Table S1. Reagents used in this study

Table S2. Primers used in this study

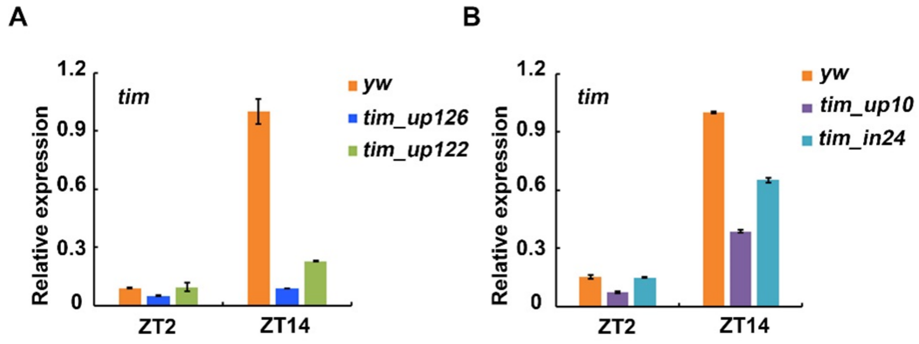

Supplemental Figure 1. qRT-PCR verification of *tim* expression in E box deletion mutants.

(A-B) Flies were entrained in LD condition for 3 days before collected at ZT2 and ZT14, RNA was extracted from heads. *tim* expression levels were determined by qRT-PCR and normalized to *RPL32*. Values are represented relative to the maximum value. Error bars represent the standard deviation of two replicates.

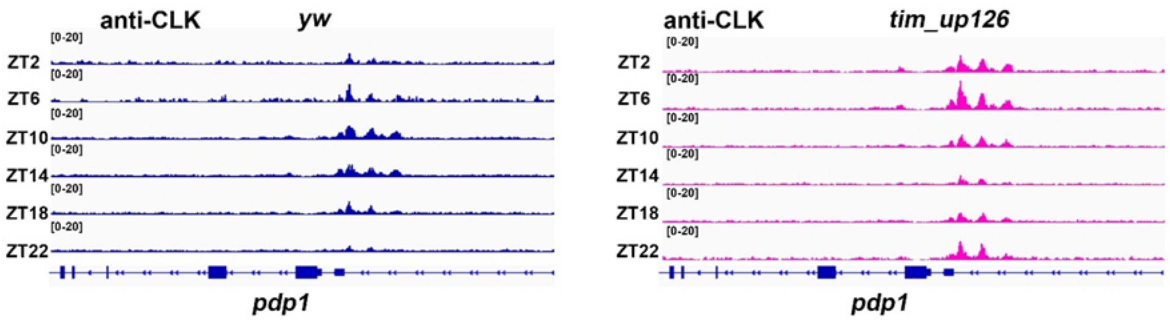

Supplemental Figure 2. TIM affects CLK DNA binding activity to regulate *pdp1* transcription.

CLK ChIP signals at the *pdp1* locus in *yw* (left panel) and *tim_up126* (right panel) across 6 time points in a 12:12 LD cycle.

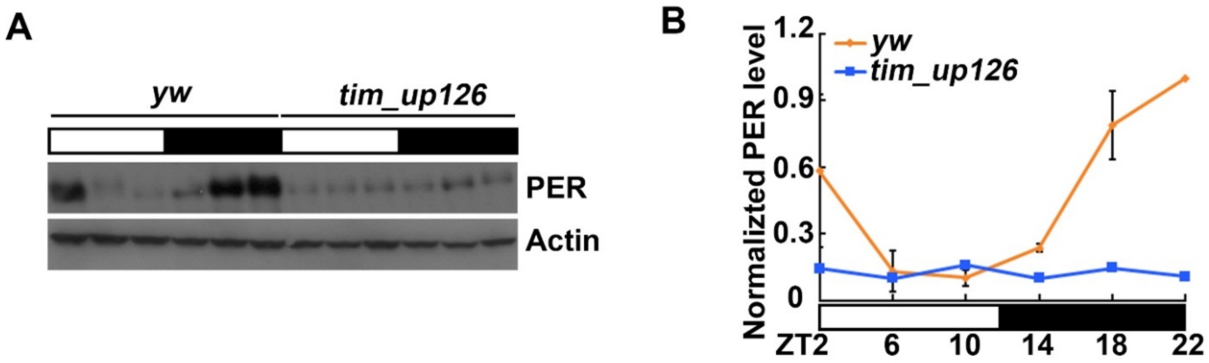

Supplemental Figure 3. PER expression is dramatically reduced and shows no robust cycling in the head of tim_*up126* under LD conditions.

(A) A representative Western blot showing PER levels in wild-type (*yw*) and *tim_up126* mutant flies.. (B) Quantification of the PER levels relative to beta-Actin in *yw* and *tim_up126* as shown in (A). Error bars represent the standard deviation of two biological replicates.

Table S1. Reagents used in this study

Reagent type (species)	Designation	Source	Identifiers	Additional information
Genetic reagent (D. melanogaster)	nSyb-Gal4	BDSC	RRID: BDSC_51635	
Genetic reagent (D. melanogaster)	Clk856-Gal4	(65)	Flybase: FBtp0069616	
Genetic reagent (D. melanogaster)	Repo-Gal4	BDSC	RRID: BDSC_7415	
Genetic reagent (D. melanogaster)	UAS-EGFP	BDSC	RRID: BDSC_5428	
Genetic reagent (D. melanogaster)	UAS-EGFP	BDSC	RRID: BDSC_56545	
Genetic reagent (D. melanogaster)	CLK-V5	(50)		
Genetic reagent (D. melanogaster)	3x Flag-CLK14.8-HBH	(66)		
Genetic reagent (D. melanogaster)	AGES system	BDSC	RRID: BDSC_92470	
Genetic reagent (D. melanogaster)	*tim_in24*	This study		

Genetic reagent (D. melanogaster)	*tim_up10*	This study		
Genetic reagent (D. melanogaster)	*tim_up122*	This study		
Genetic reagent (D. melanogaster)	*tim_up126*	This study		
Antibody	Anti-PER Rabbit polyclonal	Laboratory of Michael Rosbash		1:1000
Antibody	Anti-TIM Rat monoclonal	Laboratory of Michael Rosbash	RRID: AB_2753140	1:200
Antibody	Goat anti-rabbit polyclonal	ThermoFisher	RRID: AB_2633281	1:200
Antibody	8D12 anti-Repo	DSHB	Supernatant	1:100

Table S2. Primers used in this study

#	Purpose	sequence
gRNA-1	Intronic E-box deletion	GTCGCTGTGCGGACACGTGTGTG
gRNA-2	Intronic E-box deletion	AAACCACACACGTGTCCGCACAGC
gRNA-3	upstream E-box deletion	GTCGGCACGTTGTGATTACACGT
gRNA-4	upstream E-box deletion	AAACACGTGTAATCACAACGTGCC
tim_up122/126	forward protospacer sequence	TACACGTGAGCCGATTTCCC
*tim_up122*_reverse	*tim_up122*	CTGCCGGCGTTTGTGCGAAC
*tim_up126*_reverse	*tim_up126*	ACGTGCCGCTGCCGGCGTTTC
Rpl32-For	qRT-PCR	ATCGGTTACGGATCGAACAA
Rpl32-Rev	qRT-PCR	GACAATCTCCTTGCGCTTCT
tim-For	qRT-PCR	TCCATGAAGTCCTCGTTCG
tim-Rev	qRT-PCR	CATCGCTCACATACATTCTGG
